# Relationship of circulating *Plasmodium falciparum* lifecycle stage to circulating parasitemia and total parasite biomass

**DOI:** 10.1038/s41467-022-32996-5

**Published:** 2022-09-23

**Authors:** Michael F. Duffy, Gerry Q. Tonkin-Hill, Leily Trianty, Rintis Noviyanti, Hanh H. T. Nguyen, Janavi S. Rambhatla, Malcolm J. McConville, Stephen J. Rogerson, Graham V. Brown, Ric N. Price, Nicholas M. Anstey, Karen P. Day, Anthony T. Papenfuss

**Affiliations:** 1 Peter Doherty Institute for Infection and Immunity, Melbourne, VIC Australia; 2 Bio21 Institute, University of Melbourne, Melbourne, VIC Australia; 3 Department of Microbiology and Immunology, The University of Melbourne, Melbourne, Melbourne, VIC Australia; 4 Wellcome Sanger Institute, Hinxton, UK; 5 The Eijkman Institute for Molecular Biology, Jakarta, Indonesia; 6 Department of Medicine and Radiology, Royal Melbourne Hospital, University of Melbourne, Melbourne, VIC Australia; 7 Department of Biochemistry and Molecular Biology, University of Melbourne, Melbourne, VIC Australia; 8 The Nossal Institute for Global Health, University of Melbourne, Parkville, VIC Australia; 9 Global and Tropical Health Division, Menzies School of Health Research, Charles Darwin University, Darwin, NT Australia; 10 Centre for Tropical Medicine and Global Health, Nuffield Department of Clinical Medicine, University of Oxford, Oxford, UK; 11 Bioinformatics Division, Walter and Eliza Hall Institute of Medical Research, Parkville, VIC Australia; 12 Department of Mathematics and Statistics, University of Melbourne, Parkville, VIC Australia; 13 Peter MacCallum Cancer Centre, Victorian Comprehensive Cancer Centre, Melbourne, VIC Australia; 14 Department of Medical Biology, University of Melbourne, Parkville, VIC Australia; 15 Sir Peter MacCallum Department of Oncology, University of Melbourne, Parkville, VIC Australia

**Keywords:** Parasite biology, Parasite immune evasion

**arising from** R. Thomson-Luque et al. *Nature Communications* 10.1038/s41467-021-25062-z (2021)

We previously showed in Papua that circulating *Plasmodium falciparum* causing severe malaria are younger than those causing uncomplicated malaria^1^. Thomson-Luque et al.^2^ subsequently claimed that in ours, and other datasets, circulating parasitemia inversely correlated with estimated parasite age and that this was because the *P. falciparum* that cause severe malaria express PfEMP1s that are more cytoadherent, leading to earlier parasite sequestration in the microvasculature and reduced splenic clearance. Here, we show that in our dataset circulating parasitemia and the proportion of total parasites that are circulating do not correlate with circulating parasite age, hence our data do not support their hypothesis.

Thomson-Luque et al. confirmed our discovery that circulating *P. falciparum* parasites in severe malaria are younger than those causing uncomplicated malaria^1^ and they suggested that this difference confounded comparisons between transcriptomes of parasites causing severe and uncomplicated malaria. This is precisely the reason why we developed and applied mathematical approaches to control for parasite stage variation prior to identifying genes upregulated in severe malaria by differential gene expression (DGE) analysis. It appears that Thomson-Luque et al. missed these details which were outlined in our methods and results. Thus, they may have misinterpreted our differentially expressed geneset in their reanalysis. Thomson-Luque et al. themselves showed that the genes that we identified as upregulated in severe malaria were not expressed earlier than the genes upregulated in uncomplicated malaria (Thomson-Luque et al. Fig. 3a, b). Thus, our analysis successfully controlled for parasite stage variation which did not confound our differentially expressed geneset.

Thomson-Luque showed that the genes upregulated in severe malaria in our differentially expressed geneset were not expressed earlier than the genes upregulated in uncomplicated malaria. They claimed that this similarity in expression timing was due to a lack of difference in parasitemia between our severe malaria and uncomplicated malaria patients. In our paper we analysed 44 patient samples for our *var* gene de novo assemblies and compared these parasite densities, but only 35 of these were used for DGE due to drug treatment prior to admission or insufficient sequence coverage^1^. Parasitemias were significantly higher in the 16 severe malaria cases used for DGE (median 2.071%, IQR 0.422–12.83) than in the 19 uncomplicated malaria cases used for DGE (median 0.59% IQR 0.092–1.18) *p* = 0.0136 two-sided Mann–Whitney test *U* = 78. Thus, because we had not provided the individual parasite densities Thomson-Luque et al. understandably but incorrectly assumed the samples used for DGE also did not differ in parasitemia.

Thomson-Luque et al. used our mixture model to estimate parasite stage in multiple datasets and showed inverse correlations between these estimates of parasite age and circulating parasitemias. They inferred that sequestered parasite load correlated with circulating parasitemia and therefore correlated inversely with circulating parasite age (Thomson-Luque et al., Fig. 5). However, our data do not support these correlations. We did not provide the parasitemias for the individual patients in our publication but we have reanalysed our data and compared the proportions of parasite stages in our samples to the parasitemias. There is no correlation in our dataset between the parasitemia and the proportion of ring stage parasites (Spearman *r* = 0.2033 95% CI −0.1494 to 0.5101 *p* = 0.2415) or asexual, non-ring stage parasites estimated by our mixture model (Spearman *r* = −0.2088 95% CI −0.5143 to 0.1438 *p* = 0.2288) (Fig. 1). Consequently, in our samples parasitemia does not correlate with parasite age.

**Fig. 1:**
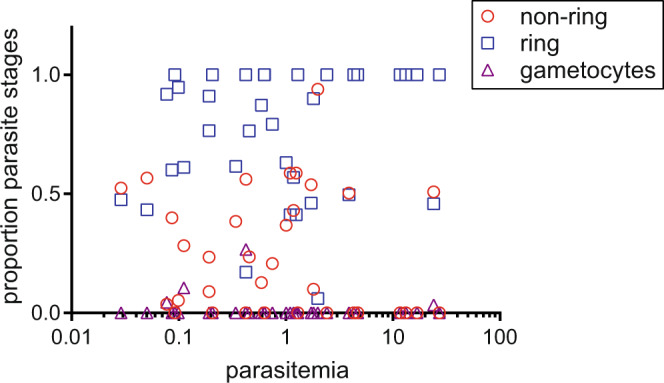
Circulating parasitemia does not correlate with circulating parasite age. Parasitemias for the 16 severe malaria samples and 19 uncomplicated malaria samples that were used for the differential gene expression analysis of the full transcriptome are plotted against the proportion of the circulating parasites estimated by our mixture model that were ring stages, asexual non-ring stages and gametocytes^1^. Source data are provided as a Source Data file.

In their Fig. 1c, Thomson-Luque et al. ranked 41 of our patient samples by the RNAseq readcounts of a single glycine tRNA ligase gene PF3D7_1420400 in lieu of the individual parasitemias. This gene is described as a housekeeping gene and its level of expression is inferred to represent parasitemia. However, in the 41 samples with adequate sequence coverage for expression analysis from Tonkin-Hill et al.^1^ there is no correlation between the levels of normalised reads of PF3D7_1420400 and parasitemia (Spearman *r* = −0.1908 95% CI −0.4687 to 0.1213, *p* = 0.2147). There is a correlation between levels of normalised reads of PF3D7_1420400 and proportions of ring stages (Spearman *r* = 0.4934, 95% CI 0.1432 to 0.6634, *p* = 0.004) or proportion of asexual non-ring stages (Spearman *r* = −0.4608 95% CI −0.6782 to −0.1693 *p* = 0.0024). That the normalised glycine tRNA ligase reads have a positive correlation with the proportion of ring stages and a negative correlation with the proportion of non-ring asexual stages suggests that the levels of glycine tRNA ligase transcripts better reflects youth of parasite than parasitemia, consistent with the majority of RNAseq datasets prepared using the same approach as our own and available on plasmodb^3–7^. Thus, the inferred relationship between raw reads of glycine tRNA ligase and circulating parasitemia in the samples of Tonkin-Hill et al. was incorrect and the observed trends in gene expression of the Tonkin-Hill et al. samples in Fig. 1c of Thomson-Luque et al. are not associated with increasing parasitemia.

Thomson-Luque et al. used staging of transcriptomes of circulating parasites to infer relationships between parasite stage and circulating parasite density and proposed that earlier sequestration occurs in high parasitemia/severe malaria. However, we present data suggesting that in our samples this was not the case using estimated total parasite biomass (*Ptot*) calculated from HRP2 levels. *Ptot* estimates both sequestered and circulating parasites^8^ and thus more directly measures the removal of parasites from circulation by cytoadhesion than inferring cytoadhesion from differences in circulating parasitemia. *Ptot* can also be affected by variation in the parasite multiplication rate, duration of infection, volume of distribution, and inter-individual variation in PfHRP2 half-life but is nonetheless a better univariate correlate with clinical outcome and prognostic indicators of severity than is peripheral blood parasitemia^8^. *Ptot* could be calculated for 21 uncomplicated and 16 severe malaria samples^1^. √Ptot was higher in severe malaria (mean ± SEM 2,132,578 ± 399,093) than in uncomplicated malaria (mean ± SEM 1,090,126 ± 133,654) in these samples (two-sided unpaired *t*-test *p* = 0.0094, *t* = 2.749, df = 35, raw Ptot were right-skewed so data were square root transformed and then normality was confirmed by D’Agostino & Pearson normality test). However, the ratio of circulating parasitemia/Ptot, i.e., a relative measure of the proportion that circulating parasites constitute of the body’s total parasite load, did not correlate with the proportion of circulating parasites that were rings (Spearman *r* = −0.0767 95% CI −0.3994 to 0.2629, *p* = 0.6520) or asexual non-ring stages (Spearman *r* = 0.0923 95% CI −0.2483 to 0.4125, *p* = 0.5871) (Fig. 2). This highlights that in our dataset the proportion of total parasites that were sequestered had no association with the stage of the circulating parasites. These results do not support the model proposed by Thomson-Luque of high parasitemia leading to severe disease due to earlier sequestration and thus younger circulating parasites, which we would expect to manifest as a lower proportion of total parasites circulating in patients with younger circulating parasites.

**Fig. 2:**
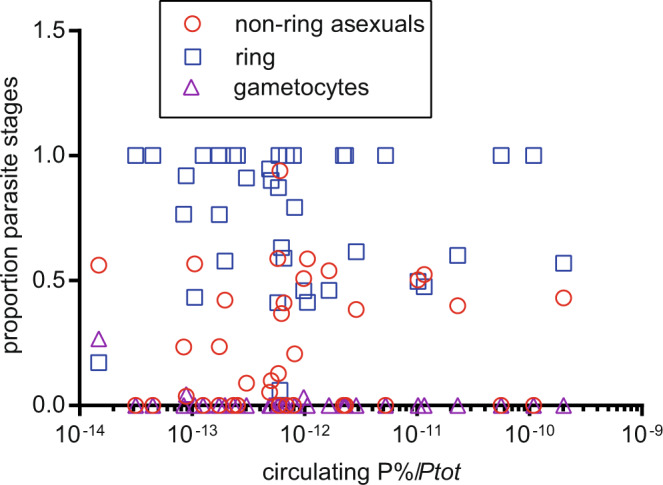
A relative measure of the proportion of total parasites that were circulating, i.e., the ratio of circulating parasitemia/calculated total parasite biomass (Ptot) does not correlate with proportions of circulating ring stage or asexual, non-ring stage parasites. Circulating parasitemia/Ptot from 37 malaria patient samples^1^ plotted against the proportion of gametocytes, asexual non-ring stage parasites and ring stage parasites estimated by our mixture model from the parasite transcriptomes of each sample^1^. Source data are provided as a Source Data file.

Our data do support part of the hypothesis of Thomson-Luque et al., notably the established role of PfEMP1-mediated sequestration in severe disease. While that was a principal focus of our study, Thomson-Luque et al. interpreted our manuscript as reporting *var* gene expression was reduced in severe cases when in fact we reported that “There was no difference between severe malaria and uncomplicated malaria in total var gene expression”^1^. Thomson-Luque et al. appear to have misinterpreted our statement that “*var* gene expression was modulated”, which referred to a histone methyl transferase involved in *var* gene silencing and switching that was downregulated in severe malaria. Thus, we were referring to potentially altered *var* gene switching or silencing, which would affect which *var* genes were expressed not the total level of *var* gene expression.

In summary, we previously reported that parasites circulating in severe malaria were younger than those in uncomplicated malaria^1^. Our interpretation of these data differs significantly from that of Thomson-Luque et al. because in our data neither parasitemia (Fig. 1) nor direct evidence of the proportion of total parasites that were circulating (Fig. 2) correlated with circulating parasite age. While the data of Thomson-Luque et al. are of potential importance in understanding pathogenesis in malaria, our data do not support the hypothesis of Thomson-Luque et al., which was based on indirectly inferring sequestered parasite load from circulating parasitemia. Both our study and that of Thomson-Luque et al. used bulk RNAseq data to estimate circulating parasite lifecycle stage distributions. This allows broad conclusions that parasite ages differ but full resolution of life-cycle stage distribution will require single-cell RNAseq analyses, which could also improve expressed *var* gene assemblies. The identification of surface expressed PfEMP1s will require development of PfEMP1 variant-specific detection reagents.

## Reporting summary

Further information on research design is available in the Nature Research Reporting Summary linked to this article.

## Supplementary information

Reporting Summary

## Data Availability

The authors declare that all data supporting the findings of this study are available within the source_data file. Source data are provided with this paper.

## Source data

Source Data
